# Anxiety and depression in children and adolescents with obesity: a nationwide study in Sweden

**DOI:** 10.1186/s12916-020-1498-z

**Published:** 2020-03-03

**Authors:** Louise Lindberg, Emilia Hagman, Pernilla Danielsson, Claude Marcus, Martina Persson

**Affiliations:** 1 Division of Pediatrics, Department of Clinical Science, Intervention and Technology, Karolinska Institutet, Blickagången 6A Novum, 141 57 Stockholm, Sweden; 2 Department of Medicine, Solna, Clinical Epidemiology Unit, Karolinska Institutet, Stockholm, Sweden; 3 Department of Diabetes and Endocrinology, Sachsska Children’s Hospital, Södersjukhuset, Stockholm, Sweden; 4 Department of Clinical Science and Education, Södersjukhuset, Karolinska Institutet, Stockholm, Sweden

**Keywords:** Obesity, Anxiety, Depression, Children, Adolescents, Epidemiology, Cohort study

## Abstract

**Background:**

Anxiety and depression are more common in children with obesity than in children of normal weight, but it is unclear whether this association is independent of other known risk factors. Interpretation of results from previous studies is hampered by methodological limitations, including self-reported assessment of anxiety, depression, and anthropometry. The aim of this study was to investigate whether obesity increases the risk of anxiety or depression independently of other risk factors in a large cohort of children and adolescents, using robust measures with regard to exposure and outcome.

**Methods:**

Children aged 6–17 years in the Swedish Childhood Obesity Treatment Register (BORIS, 2005–2015) were included (*n* = 12,507) and compared with a matched group (sex, year of birth, and area of residence) from the general population (*n* = 60,063). The main outcome was a diagnosis of anxiety or depression identified through ICD codes or dispensed prescribed medication within 3 years after the end of obesity treatment. Hazard ratios (HRs) with 95% confidence intervals (CIs) from Cox proportional models were adjusted for several known confounders.

**Results:**

Obesity remained a significant risk factor for anxiety and depression in children and adolescents after adjusting for Nordic background, neuropsychiatric disorders, family history of anxiety/depression, and socioeconomic status. Girls in the obesity cohort had a 43% higher risk of anxiety and depression compared to girls in the general population (adjusted HR 1.43, 95% CI 1.31–1.57; *p* < 0.0001). The risk in boys with obesity was similar (adjusted HR 1.33, 95% CI 1.20–1.48; *p* < 0.0001). In sensitivity analyses, excluding subjects with neuropsychiatric disorders and a family history of anxiety/depression, the estimated risks in individuals with obesity were even higher compared with results from the main analyses (adjusted HR [95% CI]: girls = 1.56 [1.31–1.87], boys = 2.04 [1.64–2.54]).

**Conclusions:**

Results from this study support the hypothesis that obesity per se is associated with risk of both anxiety and depression in children and adolescents.

## Background

The worldwide prevalence of anxiety and depression in children is estimated to 6.5% and 2.6%, respectively [1], and rates are increasing [2]. It has been reported that children with obesity are more likely to suffer from anxiety and depressive symptoms compared to peers of normal weight [3, 4], but whether obesity per se is a risk factor for these conditions is unclear. Previous research has shown bidirectional associations between obesity and anxiety/depression. However, interpretation of these results is limited by small samples [5] and weak definitions of both exposure and outcome, i.e. self-reported data on anthropometry [6, 7] and assessment of anxiety/depressive symptoms based on questionnaires [8–11]. Reliable measures of weight/height and robust definitions of anxiety and depression are required for accurate assessment of risks. Addressing the influence of potential confounders on the association between obesity and anxiety/depression is also important when analysing risks.

Neuropsychiatric disorders and low socioeconomic status (SES) are known risk factors for anxiety and depression [12–14] and are more prevalent in children with obesity [15–17]. Associations between different ethnic groups and risk of depression have also been observed, although results are inconsistent [9, 18]. Thus, it is important to consider these factors when investigating risks of anxiety and depression in children with obesity.

The primary aim of this study was to investigate whether obesity, independently of other well-established risk factors, increases the risk of anxiety or depression in children and adolescents. For this purpose, we used detailed and prospectively collected population-based data on a large group of children and adolescents with and without obesity. Anxiety and depression were ascertained by diagnoses and/or dispensed prescription of anxiolytics and antidepressants by a physician.

## Methods

### Subjects

Individuals with obesity aged 6–17 years and enrolled in the Swedish Childhood Obesity Treatment Register (BORIS) between 1 January 2005 and 30 September 2015 were included in this study. The main purpose of the register is quality assessment and long-term monitoring of obesity treatment in children and adolescents [19]. The register, which was initiated in 2005, includes information on anthropometric measures, biochemical analyses, physical activity, psychosocial situation, family history of disease, and data on current and former medical diagnoses. Treatment of childhood obesity is based on behavioural lifestyle modification. After an opt-out approval, local health care providers record weight and height data in the register during each clinical visit. The register does not include any information on children/adolescents with obesity who decline registration.

Individuals from the general population were matched to individuals in the obesity cohort according to year of birth, sex, and area of residence, by the year in which obesity treatment commenced. The area of residence was defined by the approximately 2000 districts in Sweden. Information on anthropometry was not available for individuals in the general population. Matching was performed using density matching without replacement, with five individuals from the general population per child with obesity. Using unique personal identity numbers allocated to all Swedish citizens [20], data from several national registers was linked by the governmental agencies Statistics Sweden (www.scb.se/en) and the National Board of Health and Welfare (www.socialstyrelsen.se/en). Information on SES was collected from the Longitudinal Integration database for Health Insurance and Labour Market studies, and information on anxiety and depression among the study subjects and their parents was collected from the National Patient Register and the Swedish Prescribed Drug Register.

**Fig. 1:**
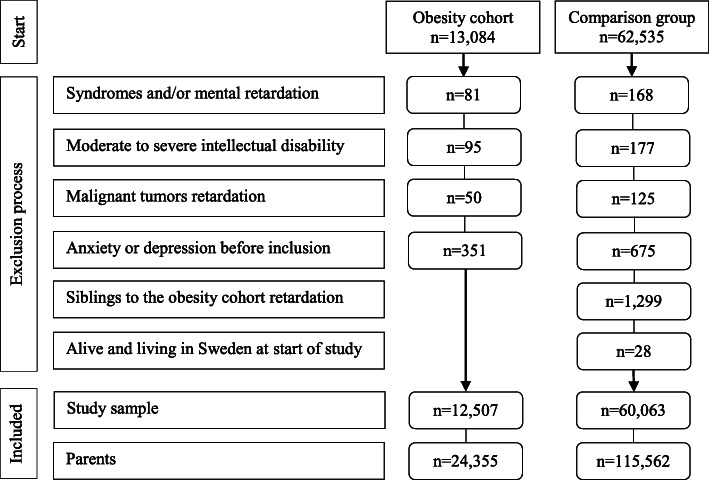
Participant flowchart

Individuals with genetic syndromes, a diagnosis of malign tumours, or moderate to severe intellectual disability were excluded from both groups (Fig. 1 and Additional file 1). In an attempt to ensure that exposure occurred before outcome, individuals with a history of anxiety or depression before the onset of obesity treatment were also excluded (Fig. 1). The regional Ethics Committee in Stockholm, Sweden, approved the study (No. 2016/922-31/1).

### Exposure variables

Information on children and adolescents in obesity treatment was collected from the Swedish Childhood Obesity Treatment Register. The degree of obesity was expressed as a body mass index standard deviation score (BMI SDS) [21]. The change in BMI SDS from the first to the last clinical visit was used to measure treatment response and categorised into four groups: good response (decrease of ≥ 0.25 BMI SDS units) [22], no response (decrease of < 0.25 to increase of < 0.25 BMI SDS units), poor response (increase of ≥ 0.25 BMI SDS units), and dropouts. Dropouts included children with 6 months or less between their first and last measurement of weight and height or with no clinical follow-up after their initial treatment visit.

### Definition of anxiety and depressive disorders

Diagnoses and information on medication, prescribed for anxiety and/or depression, were collected for individuals between 6 and 18 years of age and within 3 years after the end of obesity treatment. Diagnoses were identified by means of ICD codes according to the International Classification of Diseases 10th revision (ICD-10) or dispensed prescribed medication using the Anatomical Therapeutic Chemical classification system (ATC) (anxiety disorder: ICD-10 F40-42, ATC N05B, N05CD; depressive disorder: ICD-10 F32-F33, ATC N06A; see Additional file 1). In Sweden, diagnoses of anxiety and depression and prescriptions for anxiolytics and antidepressants are only given by physicians during inpatient or outpatient health care visits [23]. The National Patient Register contains data on clinical diagnoses from inpatient care and outpatient services with national coverage since 1987 and 2001, respectively [23]. Dispensed prescribed anxiolytics and antidepressants have been recorded in the Swedish Prescribed Drug Register since 2005 [24]. In Sweden, health care visits are free of charge until children reach 18 years of age. Since 1 January 2016, prescribed medication has been free of charge for individuals under 18 years of age and was heavily subsidised prior to that (limit of approximately 220 EUR per person per year).

### Covariates

Information on sex and Nordic background was obtained from the Swedish Total Population Register [25]. Backgrounds were defined as Nordic (child and at least one parent born in a Nordic country [Sweden, Norway, Denmark, Finland, or Iceland]) and non-Nordic (child born outside the Nordic region or born in the Nordic region, but where both parents were born outside the Nordic region). Neuropsychiatric disorders were defined as diagnosis of attention deficit disorder with or without hyperactivity (ADHD/ADD), mild intellectual disability, and/or autism spectrum disorder based on ICD-10 codes (Additional file 1). Children with ADHD/ADD were also identified using data on prescriptions of medications for these conditions (Additional file 1). Data on age and BMI SDS at the start of obesity treatment was obtained from the Swedish Childhood Obesity Treatment Register, and information on age at first anxiety and/or depressive disorder was obtained from the Swedish Prescribed Drug Register and the National Patient Register.

Family history of anxiety and depression was defined as diagnosis or at least two dispensed prescribed medications for either parent (Additional file 1). SES was estimated on the basis of level of maternal and paternal education, occupation, and income [16]. The highest attained level of education was classified as completion of compulsory education, upper secondary education, or university degree (scores 0, 1, and 2), according to the International Standard Classification of Education [26]. Annual disposable income, converted to 2015 prices using the Consumer Price Index (www.scb.se/en), was categorised into quartiles (scores 0, 1, 2, and 3). A period of at least 6 months of unemployment was defined as no occupation (score 0). Occupation included people registered as employed for most of the year in question (score 1). Data was taken from three points in the child’s life: at ages 6, 12, and 17 years. The mean of all available data points (0–6 points) for SES was calculated and categorised into four groups: low SES (0–1.5 points), medium-low SES (2–3 points), medium-high SES (3.5–4.5 points), and high SES (5–6 points). If the child had both biological and adoptive parents (obesity cohort *n* = 136, comparison group *n* = 928), data on the adoptive parent was used.

### Statistical analysis

Descriptive statistics are presented as means and standard deviations (SD) or numbers and percentiles. Group *t* tests (for continuous variables) and chi-square tests (for categorical variables) were used to estimate differences between the obesity cohort and the comparison group and between girls and boys. Age and BMI SDS at the start of obesity treatment and age at first anxiety or depressive disorder were treated as continuous variables in the analyses.

Cox proportional models were used to calculate the hazard ratio (HR) and 95% confidence interval (CI) for the outcomes. Follow-up began at the start of treatment (the date of treatment initiation for each individual with obesity was applied to their matched peers in the comparison group) and ended at first anxiety or depressive disorder, emigration, death, 18 years of age, 3 years after the end of obesity treatment, or the closing date (30 November 2018), whichever came first. Multivariate Cox proportional hazard analyses included adjustment for Nordic background, neuropsychiatric disorders, family history of anxiety/depression, and SES. Sensitivity analyses were performed, excluding children with neuropsychiatric disorders and children with a family history of anxiety/depression.

Secondary analyses were performed in individuals with obesity to investigate the impact of several covariates on the risk of anxiety or depression. All analyses were performed using SAS version 9.4 (Cary, NC, USA). Complete case analyses were performed as missing data was rare (missing SES; obesity cohort 0.6%, comparison group 1.4%).

## Results

A total of 12,507 individuals with obesity (46.9% girls) and 60,063 individuals in the comparison group (47.1% girls) were included in the study. In children with obesity, 9.7% suffered from anxiety or depression compared with 5.0% of individuals in the comparison group (*p* < 0.0001). Overall, anxiety and depression were more common in girls than in boys (7.0% vs. 4.8%; *p* < 0.0001), although boys were roughly 8 months younger than girls at first diagnosis or dispensed prescribed medication (*p* < 0.0001). Of all children with anxiety and depression, 56% had at least one parent with anxiety/depression (*p* < 0.0001). Common risk factors for anxiety and depression, e.g. neuropsychiatric disorders and low SES, were more prevalent among children and adolescents with obesity compared to the comparison group. Characteristics of the study cohort stratified by sex are shown in Table 1.

**Table 1:** Characteristics of the participants (*n* = 72,570)

	Obesity cohort		Comparison group	
	Girls, *n* = 5867	Boys, *n* = 6640	Girls, *n* = 28,270	Boys, *n* = 31,793
Nordic	3943 (67.2)	4400 (66.3)	20,010 (70.8)	22,837 (71.8)
Anxiety and/or depressive disorders^a^	681 (11.6)	532 (8.0)	1707 (6.0)	1310 (4.1)
Age at first anxiety and/or depressive disorders	14.5 (2.3)	13.9 (2.7)	14.7 (2.5)	14.0 (2.8)
Anxiety disorder^b^	518 (8.8)	403 (6.1)	1331 (4.7)	995 (3.1)
Age at first anxiety disorder	14.4 (2.4)	13.8 (2.8)	14.5 (2.5)	13.8 (2.9)
Depressive disorder^b^	426 (7.3)	297 (4.5)	966 (3.4)	634 (2.0)
Age at first depressive disorder	15.1 (1.8)	14.5 (2.4)	15.4 (1.9)	14.8 (2.3)
Neuropsychiatric disorder^c^	786 (13.4)	1412 (21.3)	1301 (4.6)	2793 (8.8)
ADHD/ADD	612 (10.4)	1107 (16.7)	1005 (3.6)	2286 (7.2)
Autism spectrum disorder	201 (3.4)	473 (7.1)	342 (1.2)	809 (2.5)
Mild intellectual disability	127 (2.2)	207 (3.1)	186 (0.7)	331 (1.0)
Parental anxiety/depression^d^	3129 (53.3)	3666 (53.3)	11,663 (41.3)	13,382 (42.1)
Mothers	2477 (42.2)	2891 (43.5)	8762 (31.0)	9916 (31.2)
Fathers	1450 (24.7)	1700 (25.6)	5224 (18.5)	6126 (19.3)
SES				
Low SES	1067 (18.2)	1253 (18.9)	3593 (12.7)	4089 (12.9)
Medium-low SES	2202 (37.5)	2513 (37.9)	8023 (28.4)	9057 (28.5)
Medium-high SES	1996 (34.0)	2248 (33.9)	10,689 (37.8)	12,182 (38.3)
High SES	568 (9.7)	590 (8.9)	5564 (19.7)	6037 (19.0)
Missing	34 (0.6)	36 (0.5)	403 (1.4)	433 (1.4)

Data is *n* (%) or mean (SD)

^a^Diagnosis of anxiety and/or depression and/or prescription of medication to treat anxiety and/or depression

^b^Diagnosis and/or prescription medication

^c^Includes ADHD/ADD, autism spectrum disorder, and mild intellectual disability in the child

^d^Includes maternal and paternal diagnosis of anxiety and depression or at least two dispensed prescriptions of anxiolytics/antidepressants

*Abbreviation*: *SES* socioeconomic status

### Risk factors for anxiety and depression

**Fig. 2:**
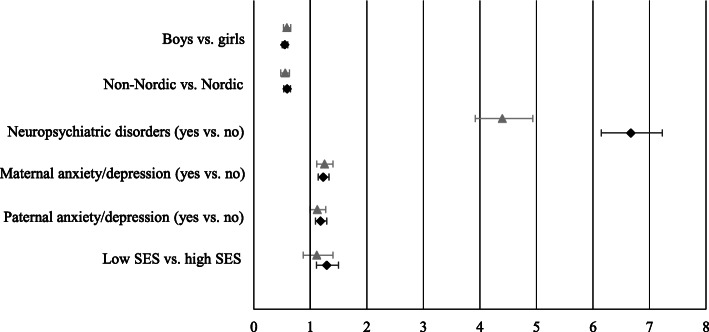
Risk of anxiety and/or depressive disorders by group. Legend: Grey triangles represent the obesity cohort, and black squares represent the comparison group. Bars represent mutually adjusted hazard ratios and 95% confidence intervals. Abbreviation: *SES* socioeconomic status

Obesity was a strong risk factor for anxiety and depression in both sexes: adjusted HR [95% CI] for girls was 1.43, [1.31–1.57], *p* < 0.0001, and for boys 1.33, [1.20–1.48], *p* < 0.0001 (Additional file 2). History of maternal and paternal anxiety/depression was of equal importance for the risk of anxiety and depression in children both with and without obesity (Fig. 2).

### Sensitivity and interaction analyses

In sensitivity analyses, excluding children with neuropsychiatric disorders and a family history of anxiety/depression, the risks for anxiety and depression were even higher in individuals with obesity compared with the adjusted risk estimates from the main analyses (Table 2). Interaction analyses were performed to explore whether the association between childhood obesity and the risk of anxiety and depression was modified by co-existing neuropsychiatric diagnoses. Adjusted analyses showed that the association between obesity in childhood and the risk of anxiety and depression was attenuated by neuropsychiatric disorders in both sexes (test for interaction *p* < 0.001, data not shown).

**Table 2:** Sensitivity analyses stratified by sex, excluding children with neuropsychiatric disorders and children with a family history of anxiety/depression

	Crude HR (95% CI); *p*	Adjusted HR (95% CI); *p*
Anxiety and/or depressive disorders		
Girls	1.61 (1.35–1.92)***	1.56 (1.31–1.87)***
Boys	2.04 (1.65–2.53)***	2.04 (1.64–2.54)***
Anxiety disorder		
Girls	1.55 (1.27–1.89)***	1.49 (1.22–1.82)***
Boys	1.86 (1.46–2.39)***	1.88 (1.46–2.42)***
Depressive disorder		
Girls	1.81 (1.42–2.30)***	1.80 (1.41–2.29)***
Boys	2.69 (1.94–3.72)***	2.68 (1.92–3.74)***

HRs show a risk of anxiety and depression for the obesity cohort versus the comparison group for girls and boys respectively. Adjusted HRs controlled for Nordic background and SES. Sample in adjusted HR; girls: obesity cohort *n* = 2432, comparison group *n* = 15,746; boys: obesity cohort *n* = 2469, comparison group *n* = 16,882. Number of total events of anxiety or depressive disorders: girls *n* = 788, boys *n* = 472. See the “Methods” section and Additional file 1 for definitions of disorders

***p < 0.0001

*Abbreviations*: *HRs* hazard ratios, *SES* socioeconomic status

### Risk factors for anxiety and depression in children and adolescents with obesity

Descriptive statistics for children and adolescents with obesity are shown in Additional file 3. At the last clinical visit, 37% of individuals who had undergone obesity treatment had morbid obesity, 48% obesity, 14% overweight, and 1% normal weight.

In both boys and girls, the risk of anxiety and/or depressive disorders increased with increasing age at the start of obesity treatment (Table 3). Significant risk factors for anxiety and depression in children and adolescents with obesity included poor treatment response, dropping out of obesity treatment, Nordic origin, neuropsychiatric disorders, and a family history of anxiety/depression (Table 3).

**Table 3:** Mutually adjusted hazard ratios with 95% confidence intervals in children with obesity, by sex, on anxiety and depressive disorders

	Anxiety and/or depressive disorders		Anxiety disorder		Depressive disorder	
	Girls	Boys	Girls	Boys	Girls	Boys
Age at start of obesity treatment	1.29 (1.25–1.33)***	1.15 (1.11–1.19)***	1.24 (1.20–1.28)***	1.11 (1.06–1.15)***	1.37 (1.32–1.43)***	1.23 (1.17–1.29)***
BMI SDS at start of obesity treatment	0.95 (0.78–1.16); 0.63	1.28 (1.04–1.57)*	0.94 (0.75–1.17); 0.56	1.20 (0.95–1.53); 0.13	1.09 (0.85–1.41); 0.48	1.24 (0.93–1.65); 0.14
Treatment response						
No response vs. good response	1.07 (0.88–1.30); 0.51	1.10 (0.87–1.40); 0.41	1.00 (0.80–1.25); 0.98	1.08 (0.83–1.41); 0.57	1.01 (0.79–1.30); 0.94	1.09 (0.79–1.51); 0.59
Poor response vs. good response	1.20 (0.93–1.55); 0.17	1.62 (1.22–2.16)**	1.11 (0.83–1.49); 0.49	1.57 (1.13–2.19)**	1.18 (0.86–1.61); 0.31	1.83 (1.27–2.65)**
Dropouts vs. good response	1.47 (1.16–1.86)**	1.46 (1.13–1.90)**	1.38 (1.06–1.80)*	1.45 (1.08–1.95)*	1.32 (0.97–1.78); 0.08	1.35 (0.94–1.93); 0.10
Nordic background (non-Nordic vs. Nordic)	0.55 (0.45–0.68)***	0.58 (0.46–0.73)***	0.56 (0.44–0.70)***	0.67 (0.52–0.86)**	0.42 (0.32–0.56)***	0.33 (0.23–0.48)***
Neuropsychiatric disorder (yes vs. no)	3.99 (3.40–4.67)***	4.07 (3.42–4.85)***	4.12 (3.44–4.94)***	3.53 (2.89–4.32)***	4.32 (3.55–5.27)***	5.33 (4.19–6.78)***
Maternal anxiety/depression (yes vs. no)	1.33 (1.14–1.56)**	1.30 (1.09–1.55)**	1.35 (1.13–1.61)**	1.35 (1.10–1.65)**	1.40 (1.15–1.70)**	1.53 (1.21–1.94)**
Paternal anxiety/depression (yes vs. no)	1.08 (0.91–1.29); 0.38	1.28 (1.06–1.54)**	1.14 (0.94–1.39); 0.19	1.32 (1.07–1.63)*	0.97 (0.77–1.22); 0.80	1.28 (1.00–1.64); 0.05
SES						
Low vs. high SES	0.82 (0.59–1.14); 0.25	0.83 (0.58–1.19); 0.30	1.02 (0.68–1.53); 0.92	0.92 (0.60–1.41); 0.70	0.70 (0.46–1.04); 0.08	0.71 (0.44–1.16); 0.18
Medium-low vs. high SES	0.85 (0.63–1.13); 0.26	0.79 (0.57–1.08); 0.14	1.08 (0.75–1.54); 0.69	0.96 (0.66–1.41); 0.84	0.62 (0.43–0.88)**	0.69 (0.46–1.05); 0.09
Medium-high vs. high SES	0.93 (0.70–1.24); 0.61	0.67 (0.48–0.92)*	1.17 (0.82–1.65); 0.39	0.80 (0.54–1.18); 0.26	0.82 (0.59–1.15); 0.25	0.66 (0.44–1.00); 0.05

***p < 0.0001; **p < 0.01; *p < 0.05

*Abbreviations*: *BMI SDS* body mass index standard deviation score, *SES* socioeconomic status

## Discussion

Results from this nationwide study in Sweden expand upon previous knowledge by demonstrating that obesity, independently of other risk factors, is associated with risk of anxiety and depression in children and adolescents. In accordance with earlier reports [15–17], we found increased rates of well-established risk factors for anxiety and depression, such as neuropsychiatric disorders and low socioeconomic status (SES), in children with obesity. However, the risk of anxiety and depression remained significantly increased in children and adolescents with obesity even after taking these factors into account.

### Comparison with other studies

Although there is a vast literature examining the association between obesity, anxiety, and depression in children and adolescents [5–11], results are diverging and comparison of results between studies is hampered by differences in definitions of exposure and outcome, as well as between study populations. Results from this study are consistent with findings from a German study reporting higher odds of physician-diagnosed anxiety and depression in children with obesity compared to children without obesity [27]. However, estimated risks in that study—as opposed to the current study—were not adjusted for migration background, family history of anxiety and depression, or SES. Another study found increased risk of depressive symptoms, but not depressive disorder, in adolescent girls with obesity compared to girls without obesity [28].

Anxiety and depressive disorders aggregate in families with a moderate heritability (40–50%) between probands and first-degree relatives [29]. In accordance with our results, previous research [30, 31] reports similar effect size of maternal and paternal depression on the risk of anxiety or depression in the offspring.

The effect of SES on the risk of anxiety and depression in individuals with obesity was limited in this study. In contrast, low parental education and income were identified as significant risk factors for depression in American adolescents. These diverging findings may reflect the fact that socioeconomic differences are greater in the USA than in Sweden, and also differences between health care systems [32].

Depression during obesity treatment presents an additional challenge for patients trying to modify their lifestyle for weight management purposes. In a clinical study of adults with obesity who were enrolled in a weight centre programme, patients with major depressive disorder lost less weight than non-depressed patients [33]. Depression is often accompanied by increased or decreased appetite [34, 35], negative thinking, low motivation, decreased self-esteem, and fatigue [35], which may adversely impact adherence to treatment. In the current study, boys—but not girls—with good treatment results were at lower risk of anxiety and depression compared to peers with poor treatment response. However, the design of the present study does not allow any conclusions with regard to causality.

### Anxiety and depression in children and adolescents with obesity

The link between obesity and anxiety/depression may be due to shared environmental, physiological, and/or genetic factors. Obesity is associated with a systemic subclinical inflammation and oxidative stress, implicated as important aetiological factors of depression [36]. Moreover, some genotypes of the FTO gene (a gene associated with fat mass and obesity) have been associated with risk of both obesity and depression [37]. Another factor to take into consideration is the effect on body weight of potential pharmacological treatments for anxiety and depression. Prescription of anxiolytics [38] and antidepressants [39] to children and adolescents has increased rapidly over the past decade. Some of these medications have been associated with risk of both weight loss and weight gain, with major variation between individuals [40]. Psychotherapy is an alternative treatment for anxiety and depression [41] and is always considered as part of the treatment of patients with these diagnoses.

There are several other potential factors that may impact the association between obesity, anxiety, and depression. Low physical activity, unhealthy diet, and sleep disturbance are a few of the factors associated with both depression and obesity [36, 42]. In addition, children with obesity are often bullied or teased about their weight, experiences that may lead to anxiety and depressive symptoms [43].

### Strengths and weaknesses of the study

Unlike previous studies [5–11, 32], we used prospectively collected information on measured weight and height and identified individuals with anxiety and depression based on diagnosis or dispensed prescribed medications. The comparison subjects were matched to children with obesity on demographic variables, and several confounders were accounted for when estimating risks. However, the present study also has some limitations. We had no information on the weight and height of the children in the comparison group, nor on the parents. The prevalence of obesity in individuals aged 7 to 17 years in Sweden is estimated to somewhere between 4 and 8% [44]. Thus, we cannot rule out that there are individuals with obesity in the comparison group. Nevertheless, if there are individuals with obesity in the comparison group, this would only lead to a bias towards null, i.e. the association between obesity, anxiety, and depression would be weakened. Furthermore, it is possible that individuals in the obesity cohort may not be representative of children with obesity in general. It may be possible that individuals receiving obesity treatment may be more motivated to make changes and more conscious of their health than peers with obesity who are not receiving treatment. Cultural differences, socioeconomic aspects, comorbidity, and previous experiences of health care are factors which may impact an individual’s choice of whether or not to seek treatment.

To limit the time between exposure and outcome, the outcome had to occur no later than 3 years after the end of obesity treatment. It is therefore possible that individuals in the obesity cohort no longer had obesity at the time of diagnosis or pharmacological treatment of anxiety and depression. However, in this study, the treatment effect was modest and the majority (85%) still had obesity at the last clinical visit.

There is also a risk of surveillance bias in the obesity cohort. It is possible that children in obesity treatment are diagnosed with anxiety or depression to a greater extent than individuals with obesity who are not in treatment or compared to peers from the general population. However, the opposite is also possible, i.e. that health care providers continue to focus on treating obesity, and not on other co-existing symptoms. Lastly, determining a diagnosis of anxiety or depressive disorder can be difficult. In Sweden, a licenced specialist in psychiatry most often provides the diagnosis. It must further be acknowledged that overall rates of anxiety and depression may be underestimated, as a large proportion of individuals suffering from these conditions do not seek medical care [45]. However, there is no reason to believe that this pattern would differ between children and adolescents with and without obesity.

## Conclusions

Results from this nationwide study support the hypothesis that obesity per se is associated with risk of anxiety and depression in children and adolescents. Anxiety and depression cause emotional and physiological stress and suffering and may also hinder obesity treatment. Thus, screening for, and treatment of, these conditions is of great importance.

## Supplementary information

**Additional file 1.** International Classification of Diseases (ICD 10th revision) codes and Anatomical Therapeutic Chemical (ATC) classification system codes used.

**Additional file 2.** Risk of anxiety and/or depressive disorders by gender. Grey triangles represent girls and black squares represent boys. Bars represent mutually adjusted hazard ratios and 95% confidence intervals.

**Additional file 3. **Descriptive statistics of children and adolescents with obesity (*n* = 12,507).

## Data Availability

The data that support the findings of this study contains sensitive information. Restrictions therefore apply to the availability of these data, which were used under licence for the current study, and so are not publicly available. According to Swedish law and the General Data Protection Regulation, the authors are not permitted to share the datasets used in this study with third parties. Given that an ethical approval is obtained, any individual may apply for data from Statistics Sweden via information@scb.se, the Swedish National Board of Health and Welfare via registerservice@socialstyrelsen.se, and the Swedish Childhood Obesity Treatment Register via http://www.e-boris.se/kontaktuppgifter/.
