# Supplementary material for: Anxiety and depression in children and adolescents with obesity: a nationwide study in Sweden
Source: BMC Med. 2020 Mar 3;18:30. doi: 10.1186/s12916-020-1498-z (PMC7033939; doi:10.1186/s12916-020-1498-z)
Supplement: Supplementary file 1 — Additional file 1. International Classification of Diseases (ICD 10th revision) codes and Anatomical Therapeutic Chemical (ATC) classification system codes used. [file 12916_2020_1498_MOESM1_ESM.docx]

| **Additional File 1.** International Classification of Diseases (ICD 10^th^ revision) codes and Anatomical Therapeutic Chemical (ATC) classification system codes used | | |
| --- | --- | --- |
| **Condition** | **ICD-10** | **ATC Code** |
| **Anxiety diagnoses** |  |  |
| *Phobic anxiety disorders* | F40 |  |
| Agoraphobia | F40.0 |  |
| Social phobias | F40.1 |  |
| Specific (isolated) phobias | F40.2 |  |
| Other phobic anxiety disorders | F40.8 |  |
| Phobic anxiety disorder, unspecified | F40.9 |  |
| *Other anxiety disorders* | F41 |  |
| Panic disorder | F41.0 |  |
| Generalized anxiety disorder | F41.1 |  |
| Mixed anxiety and depressive disorder | F41.2 |  |
| Other mixed anxiety disorders | F41.3 |  |
| Other specified anxiety disorders | F41.8 |  |
| Anxiety disorder, unspecified | F41.9 |  |
| *Obsessive-compulsive disorder* | F42 |  |
| Predominantly obsessional thoughts or ruminations | F42.0 |  |
| Predominantly compulsive acts (obsessional rituals) | F42.1 |  |
| Mixed obsessional thoughts and acts | F42.2 |  |
| Other obsessive-compulsive disorders | F42.8 |  |
| Obsessive-compulsive disorder, unspecified | F42.9 |  |
| **Dispensed prescription medication for anxiety** |  |  |
| *Anxiolytics* |  | N05B |
| *Benzodiazepine derivatives* |  | N05CD |
| **Depression diagnoses** |  |  |
| *Depressive episode* | F32 |  |
| Mild depressive episode | F32.0 |  |
| Moderate depressive episode | F32.1 |  |
| Severe depressive episode without psychotic symptoms | F32.2 |  |
| Severe depressive episode with psychotic symptoms | F32.3 |  |
| Other depressive episodes | F32.8 |  |
| Depressive episode, unspecified | F32.9 |  |
| *Recurrent depressive disorder* | F33 |  |
| Recurrent depressive disorder, current episode mild | F33.0 |  |
| Recurrent depressive disorder, current episode moderate | F33.1 |  |
| Recurrent depressive disorder, current episode severe without | F33.2 |  |
| psychotic symptoms |  |  |
| Recurrent depressive disorder, current episode severe with | F33.3 |  |
| psychotic symptoms |  |  |
| Recurrent depressive disorder, currently in remission | F33.4 |  |
| Other recurrent depressive disorders | F33.8 |  |
| Recurrent depressive disorder, unspecified | F33.9 |  |
| **Dispensed prescription medication for depression** |  |  |
| *Antidepressants* |  | N06A |
| **ADHD/ADD** |  |  |
| *Hyperkinetic disorders* | F90 |  |
| Disturbance of activity and attention | F90.0 |  |
| Hyperkinetic conduct disorder | F90.1 |  |
| Other hyperkinetic disorders | F90.8 |  |
| Hyperkinetic disorder, unspecified | F90.9 |  |
| *Psychostimulants, agents used for ADHD and nootropic* |  | N06B |
| **Mild intellectual disability** |  |  |
| Mild mental retardation | F70 |  |
| Other mental retardation | F78 |  |
| Unspecified mental retardation | F79 |  |
| **Moderate to severe intellectual disability** |  |  |
| Moderate mental retardation | F71 |  |
| Severe mental retardation | F72 |  |
| Profound mental retardation | F73 |  |
| **Autism spectrum disorder** |  |  |
| Childhood autism | F84.0 |  |
| Atypical autism | F84.1 |  |
| Asperger syndrome | F84.5 |  |
| **Malignant tumors** |  |  |
| Malignant neoplasms | C00-C97 |  |
| In situ neoplams | D00-D09 |  |
| **Genetic syndromes** |  |  |
| Noonan | Q87.1E |  |
| Prader-Willi | Q87.1F |  |
| Russel-Silver | Q87.1G |  |
| Laurence-Moon-Bardet-Biedl | Q87.8B |  |
| Down syndrome | Q90 |  |
| Turner syndrome | Q96 |  |
| Klinefelter syndrome | Q98 |  |
| Fragile X chromosome | Q99.2 |  |
