# Supplementary material for: Anxiety and depression in children and adolescents with obesity: a nationwide study in Sweden
Source: BMC Med. 2020 Mar 3;18:30. doi: 10.1186/s12916-020-1498-z (PMC7033939; doi:10.1186/s12916-020-1498-z)
Supplement: Supplementary file 2 — Additional file 2. Risk of anxiety and/or depressive disorders by gender. Grey triangles represent girls and black squares represent boys. Bars represent mutually adjusted hazard ratios and 95% confidence intervals. [file 12916_2020_1498_MOESM2_ESM.docx]

**Additional File 2.** Risk of anxiety and/or depressive disorders, by gender. Grey triangles represent girls and black squares represent boys. Bars represent mutually adjusted hazard ratios and 95% confidence intervals
