# Supplementary material for: Anxiety and depression in children and adolescents with obesity: a nationwide study in Sweden
Source: BMC Med. 2020 Mar 3;18:30. doi: 10.1186/s12916-020-1498-z (PMC7033939; doi:10.1186/s12916-020-1498-z)
Supplement: Supplementary file 3 — Additional file 3. Descriptive statistics of children and adolescents with obesity (n = 12,507). [file 12916_2020_1498_MOESM3_ESM.docx]

| **Additional File 3.** Descriptive statistics of children and adolescents with obesity (n=12,507) | | |
| --- | --- | --- |
|  | Girls, n=5,867 | Boys, n=6,640 |
| **Start of obesity treatment** |  |  |
| Age (years) | 10.1 (7.8-12.8) | 10.8 (8.4-13.2) |
| BMI SDS | 2.75 (2.49-3.07) | 2.85 (2.59-3.18) |
| Degree of obesity |  |  |
| Obesity | 3,291 (56.1) | 3,799 (57.2) |
| Morbid obesity | 2,576 (43.9) | 2,841 (42.8) |
| **Post obesity treatment^a^** |  |  |
| Treatment duration, (years)^b^ | 2.17 (1.23-3.60) | 2.24 (1.23-3.66) |
| Difference in BMI SDSᵇ | -0.17 (-0.47 to 0.06) | -0.13 (-0.43 to 0.09) |
| Treatment response (BMI SDS) |  |  |
| Good response | 1,704 (29.1) | 1,755 (26.5) |
| No response | 1,874 (31.9) | 2,220 (33.4) |
| Poor response | 436 (7.4) | 574 (8.6) |
| Dropouts (≤6 months in treatment) | 1,853 (31.6) | 2,091 (31.5) |
| Degree of obesity |  |  |
| Normal weight | 84 (1.4) | 77 (1.2) |
| Overweight | 804 (13.7) | 850 (12.8) |
| Obesity | 2,843 (48.5) | 3,180 (47.9) |
| Morbid obesity | 2,136 (36.4) | 2,533 (38.1) |
| Data is n (%) or median (IQR) |  |  |
| ^a^ Changes from the first to the last treatment visit | | |
| ^b^ Dropouts excluded | | |
| Abbreviations: *BMI SDS* body mass index standard deviation score, *SES* socioeconomic status, *IQR* interquartile range | | |
